# A primary hepatic gastrinoma accompanied by hyperplasia of multi-nodular Brunner’s glands

**DOI:** 10.1186/s40792-017-0392-1

**Published:** 2017-11-28

**Authors:** Takaomi Hagi, Yohei Hosoda, Izumi Komoto, Shinji Uemoto, Susumu Hijioka, Yoshiro Taki, Kazuhiro Nishiyama, Masayuki Imamura

**Affiliations:** 1 Department of Gastroenterological Surgery, Kansai Electric Power Hospital, 2-1-7 Fukushima, Fukushima-ku, Osaka 553-0003 Japan; 2 Department of Gastroenterological Surgery, Osaka University Graduate School of Medicine, Osaka, Japan; 3 Department of Hepato-Biliary-Pancreatic and Transplantation Surgery, Graduate School of Medicine, Kyoto University, Kyoto, Japan; 4 Department of Gastroenterology, Aichi Cancer Center Hospital, Nagoya, Japan

**Keywords:** Primary hepatic gastrinoma, Hyperplasia of Brunner’s glands, Hypergastrinemia, Selective arterial secretagogue injection test, Somatostatin receptor scintigraphy, Pancreas-preserving total duodenectomy

## Abstract

**Background:**

Primary hepatic gastrinoma causing severe ulcerogenic syndrome is extremely rare. Herein, we report a case of primary hepatic gastrinoma accompanied by hyperplasia of multi-nodular Brunner’s glands in a patient who instead, preoperatively, was suspected of having multiple duodenal gastrinomas and hepatic metastasis.

**Case presentation:**

A 57-year-old woman consulted a clinic complaining of melena, intermittent abdominal pain, diarrhea, and vomiting which had persisted for about 3 years. Six months before her presentation, she underwent segmental resection of the jejunum for acute peritonitis due to the spontaneous jejunal perforation. A blood test revealed that her serum immunoreactive gastrin (IRG) level was 12,037 pg/mL. Subsequently, she was transferred to our hospital. On computed tomography (CT), a hypervascular tumor of 23 mm in the segment 5 (S5) region of the liver was visualized. A selective arterial secretagogue injection test (SASI test) was performed twice. The first SASI test revealed that the hepatic tumor was a gastrinoma, and there was no gastrinoma in the duodeno-pancreatic region. Additionally, somatostatin receptor scintigraphy only visualized the tumor in the liver. However, the second SASI test, which was performed during the administration of a proton pump inhibitor and a somatostatin analog (octreotide acetate), revealed that there may have been gastrinomas existing not only in the liver but also in the upper part of the duodenum or the head of the pancreas. Duodenal endoscopy revealed multiple submucosal tumors in the first and the second portion of the duodenum, although a pathological examination of biopsied specimens obtained from the duodenal lesions was negative for malignant cells. Multiple endocrine neoplasia type 1 (MEN1) was excluded from her family history, and serum levels of both intact parathyroid hormone (iPTH) and calcium were within normal ranges. An anterior segmentectomy of the liver and pancreas-preserving total duodenectomy were performed on September 9, 2013. Postoperatively, her serum immunoreactive gastrin level decreased to less than 50 pg/mL. Pathological study of the resected specimens revealed a gastrinoma in the liver, but no gastrinoma in the duodenum. Interestingly, the duodenal submucosal tumor-like lesions were hyperplastic Brunner’s glands. Postoperatively, she has been well without recurrence of hypergastrinemia for 4 years.

**Conclusion:**

We report a case of primary hepatic gastrinoma in a patient who has been cured for 4 years postoperatively. The diagnosis was somewhat difficult due to the coexisting, multiple hyperplastic Brunner’s glands of the duodenum mimicking the submucosal neuroendocrine tumors, which might have developed due to long-term hypergastrinemia.

## Background

Most gastrinomas causing Zollinger-Ellison syndrome arise in the duodenum or the pancreas [1]. Primary hepatic gastrinoma is extremely rare, and the naming of it has been allowed only when a hepatic gastrinoma has been clearly proven to not be a metastasis from other intra-abdominal organs [2–4]. Duodenal gastrinoma is the first condition to be suspected among many candidates as a potential primary source of metastatic hepatic gastrinoma because duodenal gastrinomas less than 5 mm in diameter often cause hepatic metastases. These gastrinomas are difficult to identify with a routine endoscopic exam, computed tomography (CT), and even somatostatin receptor scintigraphy (SRS) [5–7]. However, they often have been correctly located with the selective arterial secretagogue injection (SASI) test [5, 7–9].

## Case presentation

**Fig. 1:**
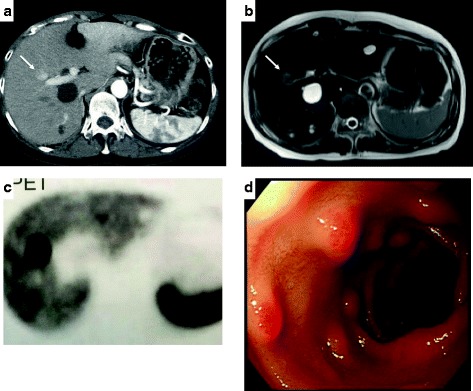
Preoperative imaging findings. **a** Abdominal contrast-enhanced computed tomography (CT) showing a hypervascular tumor in the posteroinferior segment of the right hepatic lobe (arrow). **b** Magnetic resonance imaging (MRI) showing a hypointensity tumor on T2-weighted imaging (arrow). **c** Somatostatin receptor scintigraphy (SRS) showing abnormal uptakes in the liver mass. **d** Upper gastrointestinal endoscopy showing several submucosal tumor-like lesions in the first to the second portion of the duodenum

**Fig. 2:**
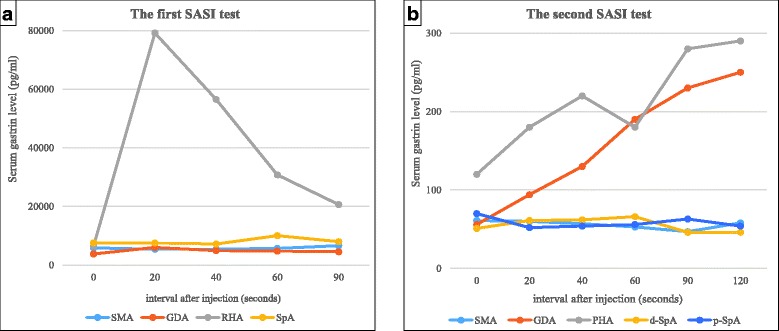
The selective arterial secretagogue injection test (SASI) test. **a** The first SASI test showed a significant elevation of the serum IRG level from 6427 to 79,160 pg/mL at 40 s after the stimulation of the right hepatic artery. **b** The second SASI test showed an elevation of the serum IRG level from 56 to 130 pg/mL at 40 s after the stimulation of the gastroduodenal artery

The patient was a 57-year-old woman who consulted a clinic complaining of melena, intermittent abdominal pain, diarrhea, and vomiting that had persisted for about 3 years. Six months before her presentation, she underwent segmental resection of the jejunum for acute peritonitis due to spontaneous jejunal perforation. A blood test revealed that her serum immunoreactive gastrin (IRG) level was 12,037 pg/mL (normal range 40–140 pg/mL), and an abdominal contrast-enhanced CT showed a hypervascular tumor of 23 mm in diameter in the segment 5 (S5) region of the liver (Fig. 1a). The tumor showed hypointensity on T2-weighted imaging and hyperintensity on diffusion-weighted imaging using abdominal contrast-enhanced magnetic resonance imaging (MRI) (Fig. 1b). A biopsy of the mass revealed a diagnosis of gastrinoma. Considering retrospectively, the previous jejunal perforation might have been caused by jejunal peptic ulcer due to excessive gastric acid secretion in Zollinger-Ellison syndrome. Based on the suspicion of metastatic gastrinoma from other intra-abdominal organs, several imaging studies were performed. On somatostatin receptor scintigraphy (SRS), only the hepatic tumor was visualized (Fig. 1c). An endoscopic ultrasound did not reveal any tumor in the pancreas. Upper gastrointestinal endoscopy revealed multiple submucosal tumor-like lesions in the first and second portions of the duodenum (Fig. 1d). A few endoscopic biopsies of the duodenal mucosa and submucosa were performed, but a pathological examination of the specimens was negative for malignant cells and could not diagnose hyperplasia of Brunner’s glands, too. The patient’s serum IRG level, despite using proton pump inhibitors (PPIs), increased to 13,339 pg/mL. During the first selective arterial secretagogue injection (SASI) test (Fig. 2a), an injection of calcium gluconate (85 mEq) was used to successively stimulate the gastroduodenal artery, the superior mesenteric artery, the right hepatic artery, and the splenic artery. Subsequently, blood samples were collected from the right hepatic vein at time points of preinjection, as well as 20, 40, 60, and 90 s after the injection of calcium solution. The serum IRG level at 40 s after the calcium stimulation rose from 6427 to 79,160 pg/mL, only after the injection into the right hepatic artery, and not after any injections into the other arteries. Therefore, it was concluded that the gastrinoma was located only in the liver.

Other endocrine examinations, such as serum levels of calcium, parathyroid hormone, prolactin, growth hormone, insulin, and glucagon, were all within normal ranges. Thus, multiple endocrine neoplasia type 1 (MEN1) was excluded. Serum levels of tumor markers were within normal ranges, except for a slight elevation in cancer antigen (CA) 19-9, at 52.2 U/mL (normal range 0–37.0 U/mL).

The results of the second SASI test, which was performed during the administration of a PPI and a somatostatin analog (octreotide acetate), were different from the first one (Fig. 2b). Specifically, the serum IRG level was elevated from 56 to 130 pg/mL at 40 s after the injection of calcium into the gastroduodenal artery, as well as into the proper hepatic artery (from 120 to 220 pg/mL), although the serum IRG level of all samples taken at the second SASI test was only about 1% of the serum IRG level of samples taken at the first SASI test (Fig. 2b).

Based on the results of the first SASI test, primary hepatic gastrinoma was initially suspected. However, at that time, we had interpreted the results of the second SASI test as that gastrinoma might be located in the upper part of the duodenum or the pancreas, because the serum IRG level was elevated from 56 to 130 pg/mL at 40 s after the injection of calcium into the gastroduodenal artery, although the serum IRG level of all samples taken at the second SASI test was only about 1% of the serum IRG level of samples taken at the first SASI test. Besides, the upper gastrointestinal endoscopy revealed multiple submucosal tumor-like lesions in the first and second portions of the duodenum which could not be differentiated from the duodenal neuroendocrine tumors endoscopically, although pathological examination of the biopsied specimens was negative for neuroendocrine tumor. On the other hand, in the pancreas, any tumor had been detected by either endoscopic ultrasound examination or CT or MRI. So, we planned to resect, with great care, the total duodenum without resecting any pancreatic tissue.

**Fig. 3:**
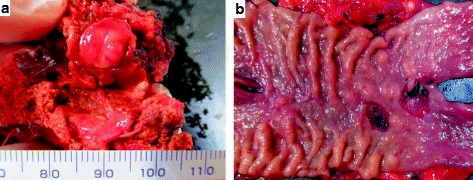
Resected specimen. **a** A solid mass, 13 mm in diameter, was observed in the liver. **b** Multiple submucosal tumor-like lesions were observed in the duodenum

**Fig. 4:**
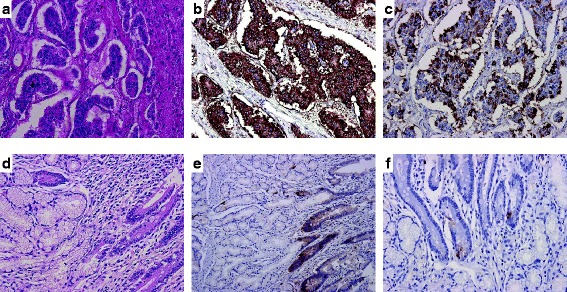
Histopathological examinations. **a** Hematoxylin and eosin staining of the liver mass showing a funicular, solid, and tubular increase of small cube-shaped, atypical cells (×200 magnification). **b** Immunostaining for chromogranin A of the liver mass was positive (×200 magnification). **c** Immunostaining for gastrin in the liver mass was positive (×200 magnification). **d** Hematoxylin and eosin staining of the duodenum showing hyperplasia of the Brunner’s glands (×200 magnification). **e** Immunostaining for chromogranin A in the duodenum was negative (×100 magnification). **f** Immunostaining for gastrin in the duodenum showing a small gathering of gastrin-positive cells (×200 magnification)

Consequently, we performed an anterior segmentectomy of the liver and a pancreas-preserving total duodenectomy. There was a solid mass measuring 13 mm in diameter in the resected liver tissue (Fig. 3a), and there were multiple submucosal tumor-like lesions in the duodenum (Fig. 3b). Histopathological examination of the liver mass revealed a gastrinoma (Fig. 4a–c). Tumor-like lesions in the duodenum were diagnosed as hyperplastic Brunner’s glands, and immunohistological staining with anti-gastrin serum did not reveal any microgastrinomas in the duodenum (Fig. 4d–f).

On the first postoperative day, the patient’s serum IRG level decreased to 26 pg/mL. Since then, her serum IRG levels have remained within the normal range. For 4 years postoperatively, the patient has had no ulcerogenic symptoms in the absence of using a PPI, and no recurrence of the hepatic tumor has been detected on CT or MRI.

## Discussion

In 1955, Zollinger and Ellison reported a syndrome in two patients involving the triad of recurrent peptic ulceration, marked gastric acid hypersecretion, and non-beta pancreatic islet cell tumors [10]. For about 30 years after this report, it had been believed that gastrinomas arose mostly in the pancreas, and sometimes blind pancreatic resections were performed in vain [10–13]. However, in 1987, Imamura et al. reported that the SASI test was useful for localization of gastrinomas, and consequently, it has become a useful guide for curative resection of gastrinomas in patients with Zollinger-Ellison syndrome (ZES) [7]. Since then, curative resection of gastrinomas has been successively performed [9, 14–17], and it has been clearly shown that ZES is caused more frequently by the duodenal gastrinoma than the pancreatic gastrinoma [17–19]. In patients with MEN1 and gastrinoma, almost all gastrinomas arise in the duodenum and are sometimes accompanied by pancreatic gastrinomas [18, 19].

Primary hepatic gastrinoma has been rarely reported [1–4]. Wu et al. reported in their study that only 8 (5.6%) of the 142 patients with ZES involved extra-pancreatic, extra-duodenal, and extra-lymphatic primary gastrinomas [20]. Gastrinomas most often metastasize to the liver and often grow larger than the primary gastrinoma. Therefore, primary hepatic gastrinoma should be diagnosed after a careful elimination of any possible metastasis from other abdominal organs.

There were a few reasons to diagnose the patient in our case as having primary hepatic gastrinoma. The first reason is that the patient had only one hepatic gastrinoma, and after resection of the hepatic tumor and the entire duodenum, the serum IRG level decreased to 26 pg/mL on the first postoperative day. Within the resected liver, there was only one tumor, which was pathologically diagnosed as a gastrinoma. Furthermore, within the resected duodenum, there were no gastrinomas, only hyperplastic Brunner’s glands. Secondarily, the duodenum was carefully examined using the anti-gastrin antibody staining and a holoblastic cleavage method, but we found no gastrinoma cells only hyperplastic Brunner’s glands. Therefore, multiple hyperplastic Brunner’s glands of the duodenum might have developed, we suspected, due to continuous, long-term hypergastrinemia of at least 3 years [21]. Thirdly, the patient’s postoperative serum gastrin levels have remained within the normal range for about 4 years, and there has been no recurrence of gastrinomas or Zollinger-Ellison syndrome.

In this case, preoperative diagnosis using the first SASI test and imaging (e.g., SRS, CT, and MRI) coincided with the final pathological diagnosis of a primary hepatic gastrinoma. However, the results of the second SASI test, which was performed under the condition of continuous administration of a PPI and octreotide acetate, did not coincide with the final diagnosis. Therefore, we now analyze the results of the second SASI test as follows: when we compare the results of two SASI tests, we see that the IRG level of the blood samples taken at the second SASI test was lower by about 1% when compared to the IRG level of the blood samples taken at the first SASI test (Fig. 2). For example, the IRG levels prior to stimulation of the arteries were between 3000 and 8000 pg/mL at the first SASI test, but they were between 50 and 120 pg/mL at the second SASI test. At the first SASI test, the maximum response of the IRG level after the injection of calcium into the proper hepatic artery was more than 70,000 pg/mL, but it was only more than 200 pg/mL at the second SASI test. Additionally, the shape of the IRG response curve after the injection of calcium into the proper hepatic artery at the first SASI test is simple and has a peak; however, the IRG response curves of the gastroduodenal artery and the proper hepatic artery at the second SASI test seem to show gradually increasing lines and have no peak. We think that these differences are because of the administration of both the PPI and octreotide acetate. At the time of the second SASI test, the hepatic gastrinoma experienced suppressed production of gastrin granules due to the administration of octreotide acetate, so the serum IRG levels at the second SASI test might have been lowered by about 1% compared to those at the first SASI test. Furthermore, at the time of the second SASI test, gastric acid secretion was suppressed due to a long-term administration of a PPI, and consequently, antral G cells were hyperfunctioning in order to stimulate parietal cells. Thus, at the time of the second SASI test, secretion of gastrin into the blood from a hepatic gastrinoma was suppressed by one-hundredth, and on the other hand, antral G cells were actively secreting gastrin into the blood. Therefore, we think that the response to the injection of calcium into the gastroduodenal artery might have been caused by the response of the hyperfunctioning antral G cells. We now conclude that the SASI test should not be performed under the administration of either octreotide or a PPI for patients with gastrinoma.

In this case, we carried out a careful histopathological examination of the duodenal specimen, which ultimately provided proof for our diagnosis.

## Conclusions

We report a rare case of a primary hepatic gastrinoma accompanied by hyperplasia of multiple duodenal Brunner’s glands. The correct diagnosis was achieved only after the anterior segmentectomy of the liver and the resection of the entire duodenum with a pancreas-preserving total duodenectomy. The presence of multiple duodenal submucosal lesions, along with the fact that the SASI test was performed under the administration of octreotide acetate and a PPI, complicated the diagnosis of the primary hepatic gastrinoma. The SASI test and SRS are useful for locating the functioning neuroendocrine tumors; however, the SASI test should not be performed during the administration of either octreotide or a PPI in patients with Zollinger-Ellison syndrome.

## Abbreviations

CT: Computed tomography
IRG: Immunoreactive gastrin
MEN1: Multiple endocrine neoplasia type 1
MRI: Magnetic resonance imaging
PPI: Proton pump inhibitor.
SASI test: Selective arterial secretagogue injection test
SRS: Somatostatin receptor scintigraphy
